# *Entamoeba histolytica* and *E. dispar* Calreticulin: Inhibition of Classical Complement Pathway and Differences in the Level of Expression in Amoebic Liver Abscess

**DOI:** 10.1155/2014/127453

**Published:** 2014-04-22

**Authors:** Cecilia Ximénez, Enrique González, Miriam E. Nieves, Angélica Silva-Olivares, Mineko Shibayama, Silvia Galindo-Gómez, Jaime Escobar-Herrera, Ma del Carmen García de León, Patricia Morán, Alicia Valadez, Liliana Rojas, Eric G. Hernández, Oswaldo Partida, René Cerritos

**Affiliations:** ^1^Departamento de Medicina Experimental, Facultad de Medicina, UNAM, Dr. Balmis 148, Colonia Doctores, 06726 México, DF, Mexico; ^2^Departamento de Infectómica y Patogénesis Molecular, CINVESTAV, 07360 México, DF, Mexico; ^3^Departamento de Biología Celular, CINVESTAV, 07360 México, DF, Mexico

## Abstract

The role of calreticulin (CRT) in host-parasite interactions has recently become an important area of research. Information about the functions of calreticulin and its relevance to the physiology of *Entamoeba* parasites is limited. The present work demonstrates that CRT of both pathogenic *E. histolytica* and nonpathogenic *E. dispar* species specifically interacted with human C1q inhibiting the activation of the classical complement pathway. Using recombinant *Eh*CRT protein, we demonstrate that CRT interaction site and human C1q is located at the N-terminal region of *Eh*CRT. The immunofluorescence and confocal microscopy experiments show that CRT and human C1q colocalize in the cytoplasmic vesicles and near to the surface membrane of previously permeabilized trophozoites or are incubated with normal human serum which is known to destroy trophozoites. In the presence of peripheral mononuclear blood cells, the distribution of *Eh*CRT and C1q is clearly over the surface membrane of trophozoites. Nevertheless, the level of expression of CRT *in situ* in lesions of amoebic liver abscess (ALA) in the hamster model is different in both *Entamoeba* species; this molecule is expressed in higher levels in *E. histolytica* than in *E. dispar*. This result suggests that *Eh*CRT may modulate some functions during the early moments of the host-parasite relationship.

## 1. Introduction

Calreticulin (CRT) is a highly conserved multifunctional protein that was originally identified as a major calcium-binding protein of the endoplasmic reticulum [1]. CRT has been detected in every eukaryotic cell, with the exception of erythrocytes. All CRT proteins contain three structural domains: a globular N-terminal domain, a proline-rich P domain, and an acidic C-terminal domain. The N-terminal domain is involved in protein-protein interactions, RNA-binding, and autoantibody binding. The P domain binds Ca^2+^ with high affinity and low capacity, while the C-terminal domain, which is the least conserved domain among CRTs, binds Ca^2+^ with low affinity [1, 2].

The role of CRT in host-parasite interactions has recently become an important area of research. CRT genes from a number of parasites (*Trypanosoma*,* Leishmania*,* Entamoeba*,* Onchocerca*,* Schistosoma*, and* Haemonchus*) have been cloned and sequenced, revealing approximately 50% identity with CRT human gene [3–8].

Although the functions of CRT are conserved in vertebrates, some CRT functions differ among parasites [9, 10]; parasite CRTs bind host C1q and inhibit C1q-dependent complement activation.* Haemonchus contortus* CRT binds host C-reactive protein and C1q; this interaction may inhibit the activation of the classical complement pathway [11]. The ectoparasite* Amblyomma americanum* secretes CRT during feeding, suggesting that the anticoagulant ability of CRT may prevent blood clotting and permit the parasite to feed on the host or induce host antiparasite responses [12]. The presence of CRT in the penetration gland cells of* Schistosoma* suggests that this molecule may be important for the host skin penetration [13].

Among protozoan parasites, the binding and inhibition of human C1q by CRT have been demonstrated in both* Trypanosoma cruzi* and* T. carassii*.* T. cruzi* and* T. carassii* CRT (*Tc*CRT) bind human or fish C1q, respectively, and specifically inhibit the classical complement pathway. This suggests an evolutive conserved interaction between CRT and C1q [14, 15].

Previously, we reported the presence of CRT in* E. histolytica* (*Eh*CRT). This protein induces an important immunogenic response in the human host. More than 90% of patients with amoebic liver abscess (ALA) develop high levels of serum antibodies against* Eh*CRT [16]. We also reported the cloning of CRT gene in* E. histolytica* and the preparation of monospecific antibodies against recombinant CRT (r*Eh*CRT); the immunohistochemical assays on trophozoites show that* Eh*CRT is located in the cytoplasmic vesicles and in vesicles in close contact with the inner cytoplasmic membrane. In histopathological studies, on sections of experimental ALA in hamsters,* Eh*CRT was clearly detected into the trophozoites and seems to be neither exposed in the surface of trophozoites nor exported into the hepatic tissue [8]. The binding of C1q to CRT in the surface of* E. histolytica* trophozoites has been recently reported after its activation in cell-to-cell interaction with Jurkat cells; authors mention that during erytrophagocytosis the CRT is located in the surface of trophozoites and in the phagocytic cups [17]. CRT in the surface of apoptotic human cells seems to function as a receptor for C1q allowing the phagocytosis of damaged cells. More so, the overexpression of crt gene is related to the presence of apoptosis inductors [18].

In mammals, translocation of CRT from the RE to the membrane can be mediated by the vesicular transportation from the RE to the Golgi, mediated by the SNARE-dependent fusion of exocytic vesicles with plasma membrane. Other possible mechanisms of translocation of CRT to the plasma membrane could be mediated by the ERP57 chaperone protein, albeit this mechanism is not yet totally demonstrated [19].

One of the indicators of virulence of* E. histolytica* trophozoites that has been cited over the years [20, 21] is resistance to the lytic action of human serum. The referred capacity of CRT to bind host C1q observed in some parasites has been considered as an evasion mechanism of the host immune response, impairing the lytic action of complement. In the case of* E. histolytica*, it is possible that resistance of virulent trophozoites to the lyses of human serum could be mediated by the C1q binding capacity of* Eh*CRT.

In the present work, we tested the human C1q binding capacity of recombinant* Eh*CRT and native CRT in an ELISA system in both pathogenic* E. histolytica* and nonpathogenic* E. dispar* species. We also demonstrated that CRT and C1q colocalize in the cytoplasmic vesicles and those near the surface membrane of previously permeabilized trophozoites. Besides, we tested the capacity of recombinant* Eh*CRT to bind human C1q and, as a consequence, be able to inhibit the classical complement pathway* in vitro.* Results suggest a clear amoebicidal activity of human serum against trophozoites that can be inhibited indistinguishably in presence of recombinant or native* Eh*CRT; the interaction of CRT-C1q evaluated was equal for both species of* Entamoebas*.

## 2. Material and Methods

### 2.1. Production of Recombinant* Eh*CRT

Full-length r*Eh*CRT and N- and C-terminal-domain proteins were expressed and purified as previously described [8, 22]. Briefly, the plasmid pBluescript-KS+ (pbKS+) was used to clone PCR products. We obtained three clones, which we refer to as pb-*Eh*CRT, pb-*Eh*CRT-N, and pb-*Eh*CRT-C. These recombinant plasmids were subcloned into the prokaryotic expression vector pProEX HT-b (Gibco Life Technologies, Grand Island, NY, USA) to express the CRT constructed in fusion with a six-histidine tag on the NH_2_ end. Competent* Escherichia coli* BL21 cells were transformed with one of the recombinant plasmids. The expression of recombinant proteins r*Eh*CRT, r*Eh*CRT-N, and r*Eh*CRT-C was induced with a final concentration of 1 mM isopropyl-*β*-D-thiogalactoside (IPTG). The QIAexpressionist system (Qiagen, Valencia, CA, USA) was used to purify recombinant proteins.

The cells were harvested by centrifugation at 3000 ×g for 12 min, and the bacterial pellet was resuspended in 5 mL of lysis buffer (8 M urea, 0.1 M NaH_2_PO_4_, and 0.1 M Tris-HCl, pH 8.0). The lysate was added to a 50% suspension of Ni-NTA agarose (Qiagen). The mixture was filtered through a filtration column (Qiagen), and the recombinant proteins were eluted with 8 M urea buffer, pH 4.5. The selected fractions were dialysed against 19 mM phosphate-buffered saline (PBS) to eliminate the urea.

### 2.2. Purification of Native* Eh*CRT and* Ed*CRT

Specific anti-r*Eh*CRT IgG antibodies were obtained previously [8] and were used to purify native* Eh*CRT and* Ed*CRT by affinity chromatography. 20 mg of IgG anti-r*Eh*CRT was bound to a Sepharose 4B column (Sigma Chemical Co., St Louis, MO, USA). A membrane-enriched* E. histolytica* or* E. dispar* extract was obtained as previously reported [23]. A 10 mg quantity of the respective antigen was applied to the column and incubated for 1 h. The column was washed with PBS, pH 7.5. This bound protein was eluted with 0.5 M glycine, pH 4.5, and 1 mL fractions were collected into 100 *μ*L of 1.0 M Tris-HCl, pH 8.5, to neutralize the acidity of the elution buffer to preserve activity. Protein concentrations were determined using a Bradford Assay kit (Bio-Rad, Hercules, CA, USA).

### 2.3. Isolation of Human Lymphocytes

Peripheral mononuclear blood cell (PMBC) was isolated from fresh human blood obtained in heparinized tubes from human volunteers. Whole blood was centrifuged by gradient of Ficoll-Hypaque (Gibco BRL); the PMBC was separated and washed three times with PBS and used immediately for assays of interaction with trophozoites of* E. histolytica* or* E. dispar* (1 : 6 ameba/lymphocytes).

### 2.4. Interaction of* Eh*CRT or* Ed*CRT with Human C1q

Microtiter plates (EIA/RIA strip, Costar, Cambridge, MA, USA) were coated overnight at room temperature (RT) with 50 *μ*L of 0–200 *μ*M of full-length recombinant r*Eh*CRT, r*Eh*CRT-N, or r*Eh*CRT-C or native n*Eh*CRT or n*Ed*CRT suspended in 0.1 M Na_2_CO_3_, pH 9.6. Each step was followed by three washes with 0.5% Tween 20/PBS. Nonspecific binding sites were blocked with 3% PBS/BSA, for 2 h at 37°C. After washing, 50 *μ*L of a 1 : 10 dilution of NHS in PBS was added to each well and incubated for 2 h at 37°C. The plates were washed as before, and 50 *μ*L of mouse anti-human C1q (1 : 500) (Santa Cruz Biotechnology, Santa Cruz, CA, USA) was added to each well and incubated for 2 h at 37°C. The plates were washed again as before, and the antigen-antibody reaction was detected by incubation with HRP-conjugated goat anti-mouse IgG (1 : 1000) for 2 h at 37°C. The reaction was developed by the addition of 200 *μ*L of* ortho-*phenylenediamine phosphate (OPD) (10 mg/mL), and the absorbance was measured at 490 nm in a microplate reader (ELx800, BioTek Instruments, Winooski, VT, USA).

### 2.5. Inhibition of C1q-Dependent Haemolytic Assays

For classical pathway complement activation, sheep red blood cells (SRBCs) were sensitized with rabbit anti-SRBC (1 : 400) (antibody ab50676, Abcam, Cambridge, MA, USA). A 500 *μ*L aliquot of 10^7^ antibody-sensitized erythrocytes (EAs) was incubated with normal human serum (NHS) diluted 1 : 10 in isotonic veronal-buffered saline containing 0.1 mM CaCl_2_, 0.5 mM MgCl_2_, 0.1% gelatine, and 1% glucose (GVB^++^) in a final volume of 1000 *μ*L as a positive control. To assess the inhibition of complement activation, the NHS (1 : 10) was preincubated with 2 *μ*g of native n*Eh*CRT or n*Ed*CRT, then added to EAs, and incubated for 1 h at 37°C. After the addition of 1 mL of cold GVB^++^, intact cells were centrifuged at 400 ×g for 15 min. Haemoglobin in the supernatant was measured at 550 nm with a DU-650 Spectrophotometer Beckman (Beckman, Danvers, MA, USA). Total haemolysis (100%) was measured by treating EAs with water. Background spontaneous haemolysis (0%) was determined by incubating EAs without serum. Haemolytic activity is expressed as a percentage of total haemolysis.

In a similar assay, 10^7^ EAs were incubated with C1q-depleted human serum (Calbiochem, a division of Merck KGaA, Darmstadt, Germany) and then added to 2 *μ*g of human C1q (Sigma). Haemolysis was calculated as before. To overcome the inhibitory effect of* Eh*CRT on the classical complement pathway, we used IgG anti-*Eh*CRT produced in mice.

### 2.6. Amoebicidal Activity of Human Serum

Axenic trophozoites of* E. histolytica*,* E. dispar*, or a virulent strain of* E. histolytica*, newly recovered from hamster livers [22], were harvested by centrifugation at 500 ×g for 10 min, washed twice with PBS, counted, adjusted to a cell density of 2 × 10^5^, and incubated with TYIS-33 medium [24] added with (10, 20, 40, and 60%) NHS. The mixtures were incubated at 37°C for 15, 30, and 60 min; viability of trophozoites was estimated through the 2% trypan blue exclusion technique [25]; live trophozoites were counted in a haemocytometer. Cell counts were expressed as a percentage of dead cells. Heat-inactivated human serum was used as a negative control. To evaluate the inhibition of lyses due to interaction of* Eh*CRT with the human C1q, 10 *μ*g of r*Eh*CRT was added to NHS incubating during 10 min; thereafter, the mixture was added to the trophozoites suspension. Lyses percentage was defined as the decrease in viable trophozoites in the presence of NHS compared with the heat-inactivated human serum control. Values were calculated as follows: (number of viable cell in control − number of viable cell in the presence of NHS)/viable cell in control) × 100. Results are the mean of three independent experiments with each* E. dispar* or* E. histolytica* species or virulent strain of* E. histolytica*.

### 2.7. Human C1q and* Eh*CRT/*Ed*CRT Colocalization

Trophozoites of* E. histolytica* or* E. dispar* were grown under axenic conditions using TYIS-33 or TYIS-2 [24], respectively, for 48 h. After incubation, the trophozoites were allowed to adhere to sterile glass cover slips for 2 h at 37°C and then fixed with 3.5% paraformaldehyde/PBS. Thereafter, cells were permeabilized or not with 0.1% (v/v) Triton X-100 and blocked with 3% BSA. Trophozoites were then incubated with 4 *μ*g of C1q for 30 min. The slides were washed several times with PBS and incubated for 1 hr with specific rabbit anti-*Eh*CRT (1 : 40 dilution) and mouse anti-human C1q antibodies (1 : 40). Thereafter, a mixture of secondary antibodies was used to reveal the antigen-antibody reactions (Alexa Fluor Cy5 goat anti-rabbit IgG and Alexa Fluor 488 goat anti-mouse IgG, both 1 : 100) (Molecular Probes, Invitrogen, Eugene, OR, USA).

In a similar assay, trophozoites were incubated with NHS (1 : 10) or human peripheral mononuclear blood cells (PMBC) (1 : 6, trophozoites/lymphocytes ratio) for 60 min before incubation with 4 *μ*g of C1q and finally processed for immunohistochemical assay as previously described [5]. Samples were examined by confocal microscopy (DM1RE-2, Leica Mikrosysteme, Wetzlar, Germany) using appropriate fluorescence emission filters. Images (*z*-series) were acquired with image-processing software (Leica, LCS Lite Profile Pro) using 0.5 *μ*m steps. The images correspond to the maximum-intensity projection of the *z*-series.

### 2.8. Experimental Amoebic Liver Abscess

Experimental acute ALA was produced in 100 g hamsters following a technique described by Tsutsumi et al. (1984) [26]. Briefly, 2.5 × 10^5^ or 2 × 10^6^ axenic trophozoites of* EhVIR* (newly recovered from hamster liver) or* E. dispar*, respectively, were inoculated into the portal vein of anesthetized hamsters. After 5, 15, and 30 min and 1, 3, and 9 hours, animals (5 hamsters at a time) were euthanized by an anaesthesia overdose. The liver was removed and fixed in 4% paraformaldehyde in PBS, followed by dehydration and paraffin embedding. Serial sections of 6 *μ*m thickness were obtained and deparaffinized from tissue blocks; lesions and trophozoites were identified by hematoxylin/eosin stain. The sliced sections were used for immunohistochemical and reverse transcriptase real-time PCR (qRT-PCR) assays.

The institutional committee previously approved protocols for animal care. The institution fulfils all the technical specifications for the production, care, and use of laboratory animals and is certified by a National Law (NOM-062-ZOO-1999). All hamsters were handled according to the guidelines of the 2000 AVMA Panel of Euthanasia.

### 2.9. Immunochemical Detection of* Eh*CRT and C1q in Amoebic Liver Abscess Lesions

Selected samples were blocked with 3% PBS/BSA solution and reacted with specific mouse anti-*Eh*CRT antibody diluted 1 : 50 and in another slice with mouse anti-human C1q antibody (1 : 20); thereafter, slices were incubated at 4°C overnight. Antigen-antibody reaction was detected using 1 : 500 dilution of goat anti-mouse IgG antibody coupled to alkaline phosphatase (Zymed Laboratories, San Francisco, CA, USA); NBT/BCIP substrate (Roche Diagnostics GmbH; Mannheim, Germany) was used as the chromogen. Monoclonal mouse IgG_1_ antibody against* Aspergillus niger* glucose oxidase was used as the negative control (clone DAK-GO1, code number X09931, Dako, Glostrup, Denmark). To avoid cross-reaction with CRT from hamster hepatic tissue, anti-*Eh*CRT antibodies were adsorbed with a lyophilized extract of hamster liver. The samples were counterstained with aqueous eosin.

### 2.10. Relative mRNA Quantification of* Ed*CRT and* Eh*CRT by qRT-PCR

The detection of CRT mRNA was carried out using a two-step* in situ* RT-PCR procedure as previously reported with some modifications [5]. Previously selected hamster liver tissue sections (3 sections after intraportal inoculation) were pretreated with 0.5 *μ*g/*μ*L proteinase K (Sigma Aldrich, St. Louis, MO, USA) and with 1 U/sample of DNase I, RNase-free (Roche Diagnostics GmbH, Mannheim, Germany). After washing with DEPC-treated water, reverse transcription was performed using SuperScript II reverse transcriptase following the manufacturer's specifications (Invitrogen, Carlsbad, CA, USA). Slides were incubated at 42° C for 2 h in a sealed humidified chamber.

The relative quantification (RQ) of the investigated samples by real-time PCR was performed using the previously synthesized cDNA in the* in situ* RT assays. For this purpose, a 7300 Applied Biosystems apparatus (Applied Biosystems, Carlsbad, CA, USA) and the Quantitect SYBR green PCR kit were used (Qiagen, Valencia, CA, USA).

qPCR was performed for 60 cycles of a 3-step PCR, including 10 seconds of denaturation at 95°C, a 30 sec primer-dependent annealing phase at 58°C, and a 10 sec template-dependent elongation at 72°C. The amplification of each template was performed in duplicate in one PCR run. The differential expression of the investigated genes was calculated as the normalized ratio to* Eh*β**-actin.

Results of the threshold cycle (Ct indicates number of cycles to which the amplified product is detected) dates were exported to an Excel sheet to calculate gene expression levels (RQ) using 2^−ΔΔCt^ method described by Livak and Schmittgen (2001) [27].

### 2.11. Statistical Analysis

All values are expressed as the mean ± SD of at least three independent experiments. Statistical significance was determined with unpaired Student's *t*-test between each condition used (control against problem), and for comparisons of multiple groups with one-way analysis of variance (ANOVA), differences were considered statistically significant when *P* values were <0.05.

## 3. Results

### 3.1. CRT Binds Human C1q

**Figure 1:**
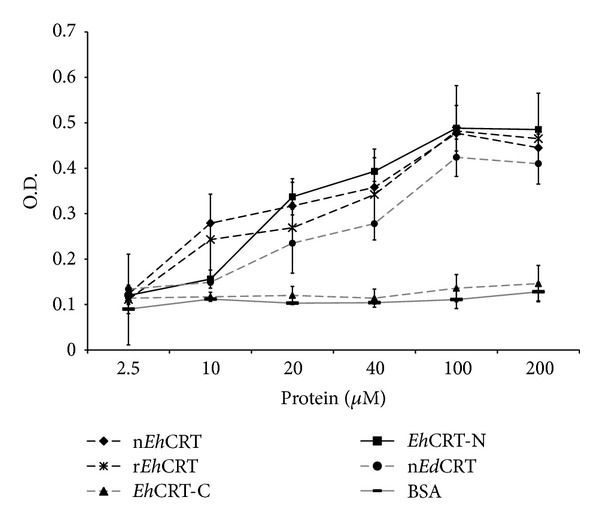
*Eh*CRT/*Ed*CRT interaction with human C1q (ELISA). Microtiter wells were coated with 2.5 to 200 *μ*M of the* Eh*CRT or* Ed*CRT and incubated with 1 : 10 diluted normal human serum supplemented with 4 *μ*g of human C1q; the interaction of* Eh*CRT-C1q was revealed using an anti-human C1q monoclonal antibody produced in mice and then an anti-mouse IgG produced in goat conjugated to peroxidase. Values are the mean of three different assays performed in triplicate ± SD.

To assess the interaction between* Eh*CRT and human C1q, a direct binding ELISA was conducted.  Figure 1 shows data of the interaction assay between r*Eh*CRT (full-length molecule, N-terminal binding domain, or C-terminal binding domain), n*Eh*CRT or n*Ed*CRT, and human C1q. Differences observed in binding between the native* Eh*CRT or* Ed*CRT and the full-length r*Eh*CRT or r*Eh*CRT-N were not statistically significant. The interaction was dose dependent and saturable; the maximum absorbance was obtained when 100 *μ*M of CRT was used; the OD remained constant in the presence of larger quantities of CRT. In contrast, the r*Eh*CRT-C-terminal protein did not bind to human C1q.

### 3.2. C1q-Dependent Haemolytic Assays

**Figure 2:**
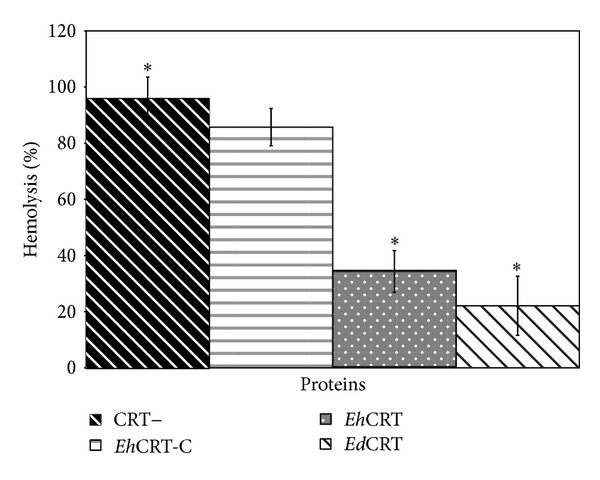
*Eh*CRT inhibits classical pathway-mediated hemolysis. Different proteins of (CRT+)* Eh*CRT or* Ed*CRT; (CRT−) BSA or* Eh*CRT-C was added to 1 : 10 dilution of NHS (as source of C1q), incubated 30 min at 37°C, and then added to 10^8^ cell/mL of EA; the mixtures were incubated for 60 min at 37° C. After centrifugation, the OD (550 nm) of the supernatants was measured. The percentage of lyses was calculated using as reference the 100% lyses of erythrocytes in water. Values are the mean of three independent experiments ± SD. Differences between groups ∗ were compared through ANOVA test detecting statistical significance (*P* = 0.05).

The ability of* Eh*CRT to inhibit the activation of the classical complement pathway by binding to C1q was tested in a simple assay of inhibition of the haemolysis of SRBCs previously sensitized with antibody (EAs). Human serum was used as the source of C1q. The optimal dilution of the human serum was determined previously by a complement titration curve (data not shown); the optimal dilution of serum was 1 : 10.  Figure 2 shows the values of inhibition of the activation of the classical complement pathway assays, in the presence of different concentrations of n*Eh*CRT or n*Ed*CRT. Both proteins inhibited the lysis of EAs in a dose-dependent manner as shown by the decrease in haemolytic activity (34–22% of baseline). By contrast, in the control assay (without CRT−) or in case we use the recombinant protein* Eh*CRT-C, there was no significant decrease in haemolytic activity (95–86% of baseline) due to the absence of C1q binding site.

**Figure 3:**
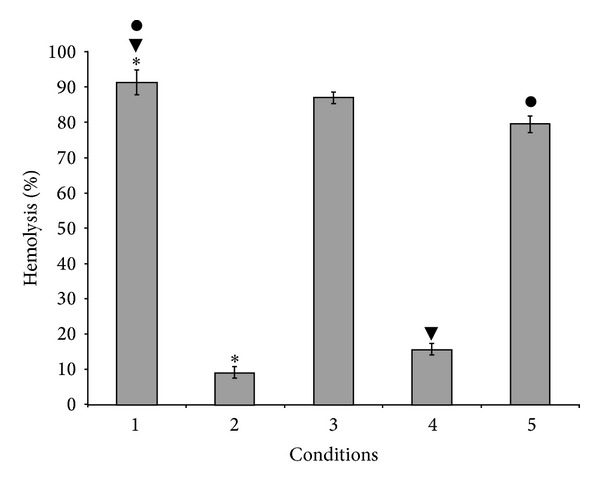
*Eh*CRT-human C1q on the activation of classical complement pathway: hemolysis assay. 1: corresponding to NHS (positive control); 2: human C1q-depleted serum (NHSC1q^−^); 3: (NHSC1q^−^) + C1q; 4: (NHSC1q^−^) + C1q +* Eh*CRT; 5: (NHSC1q^−^) + C1q +* Eh*CRT + IgG anti-*Eh*CRT. Assays were performed in triplicate; values are the mean of three different experiments ± SD. Differences between groups ∗, ●, and ▼ were compared through ANOVA test. Statistical significance (*P* = 0.012).

To confirm that this activity is the result of the interaction of human C1q with* Eh*CRT or* Ed*CRT, we used C1q-depleted human serum; when this serum was added to human C1q in the presence of* Eh*CRT, haemolysis was inhibited. Moreover, when* Eh*CRT was pretreated with anti-CRT antibodies,* Eh*CRT could not bind to C1q, and the activation of the classical complement pathway was restored (Figure 3).

### 3.3. Amoebicidal Activity of Normal Human Serum

**Figure 4:**
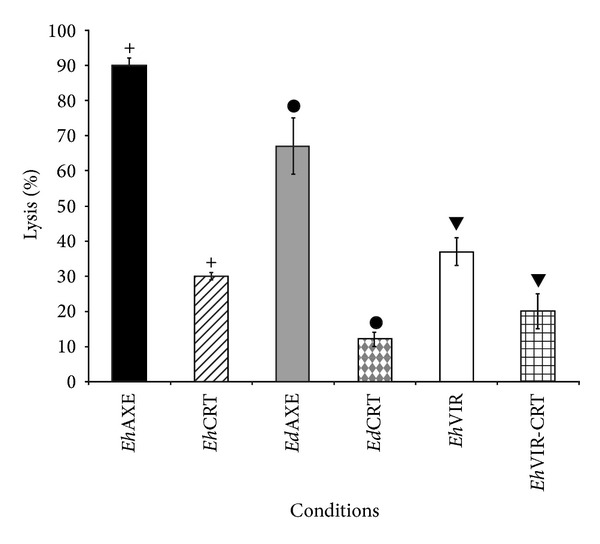
Amoebicidal activity of human serum. Trophozoites of* E. histolytica* and* E. dispar* were harvested by ice bath and then centrifuged at 500 g for 10 min, washed, counted, adjusted to a cell density of 2 × 10^5^, and incubated with TYIS-33 medium added with 40% NHS at different times; viability was assessed by trypan blue exclusion technique. To estimate the inhibition of lyses due to interaction of* Eh*CRT-C1q, 10 *μ*g of r*Eh*CRT was added to NHS incubating during 10 min, and the mixture was then added to trophozoites suspension. The percent of lyses was defined as the decrease of trophozoites viability in the presence of NHS compared with the heat-inactivated human serum control. Differences between groups** +**, ●, and ▼ were compared through Student's *t*-test detecting statistical significance (*P* < 0.05).

To test that human serum is indeed harmful to trophozoites through the action of serum complement, we previously titre the NHS; 40% of NHS was the optimal concentration to obtain reproducible results; proper time for interaction with trophozoites was 60 min. In these conditions, axenic* E. histolytica* HM1:IMSS (nonvirulent) and axenic* E. dispar* SAW760 strain are both susceptible to lysis by complement; however,* E. dispar* SAW760 is more susceptible than the virulent strain of* E. histolytica;* this strain showed a clear resistance to lyses (37%) (*P* < 0.005). The resistance of trophozoites to the lyses mediated by the r*Eh*CRT-C1q binding is shown in Figure 4. Moreover, there is a clear reduction in the values of lyses of trophozoites in the presence of r*Eh*CRT preincubated with NHS (40%). Differences with respect to controls (NHS without r*Eh*CRT) were statistically significant (*P* < 0.004).

### 3.4. Human C1q and* Eh*CRT/*Ed*CRT Colocalization

**Figure 5:**
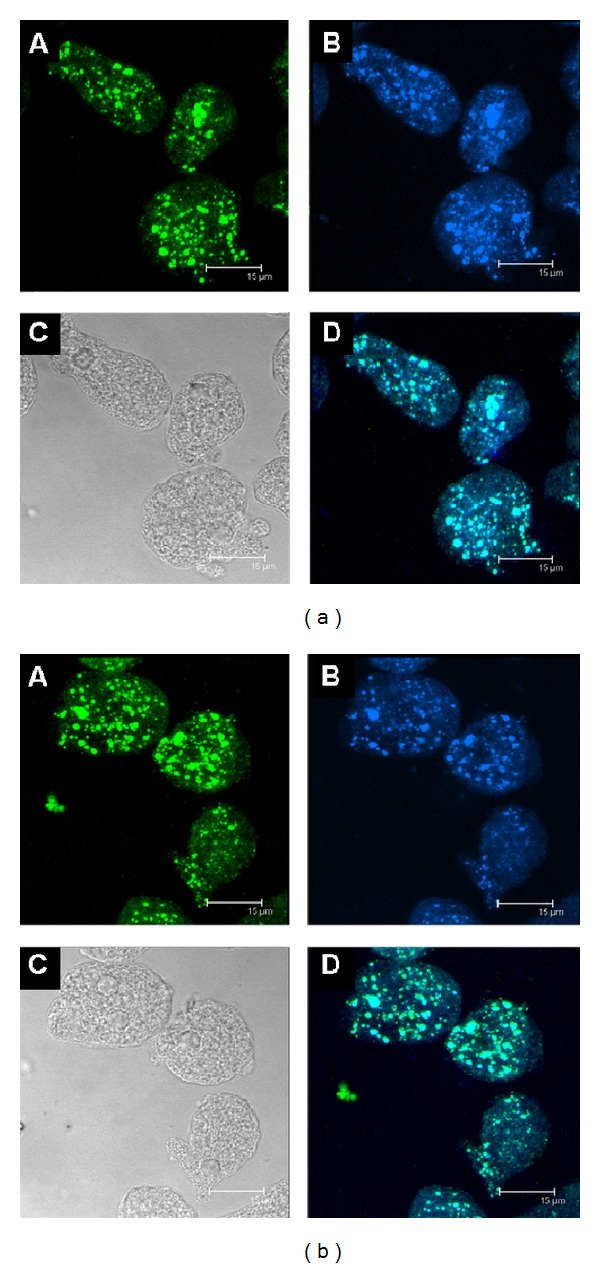
Confocal microscopy assay: colocation of* Eh*CRT or* Ed*CRT and human C1q. Panels (a) and (b) represent different patterns of immunodetection when using* E. histolytica* or* E. dispar* trophozoites, respectively; A: rabbit anti-*Eh*CRT and Alexa Fluor 350-conjugated secondary antibody; B: trophozoites reacted with mouse anti-human C1q and with anti-mouse Alexa Fluor 488; C: representing the differential interference contrast (DIC); D: colocation of* Eh*CRT with the C1q human protein (Channel Mergin). The micrographs showed the maximal projection of the *z*-series. Scale bar represents 15 *μ*m.

**Figure 6:**
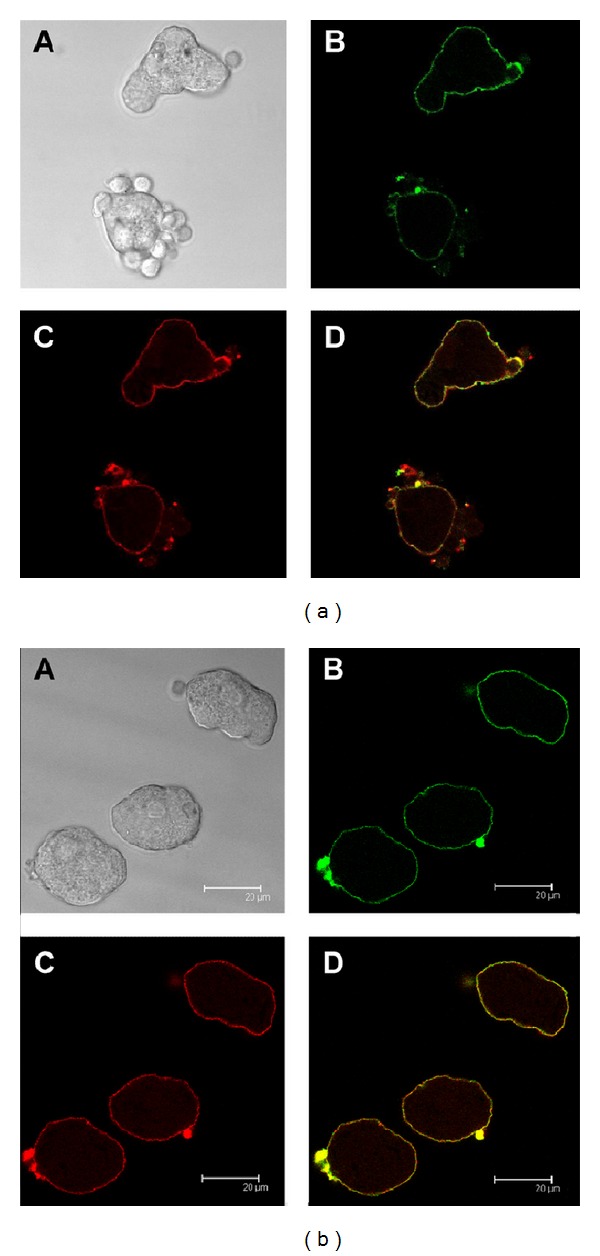
Colocalization of* Eh*CRT or* Ed*CRT and human C1q after interaction with PMBC. C1q and* Eh*CRT or* Ed*CRT colocalization by confocal microscopy of (a)* E. histolytica;* (b)* E. dispar* trophozoites. Trophozoites were grown under axenic conditions and incubated during 30 min with PMBC; thereafter, C1q was added and incubated for 30 min. Then, the mixture was added with specific primary antibodies, anti-rabbit* Eh*CRT IgG and mouse anti-human C1q IgG, respectively. The reaction was revealed with Alexa Fluor 555 goat anti-rabbit IgG and Alexa Fluor 488 goat anti-mouse IgG. The micrographs show the maximal projection of the *z*-series. Scale bar represents 20 *μ*m. A: phase contrast microscopy; B:* Eh*CRT (red); C: C1q (green); D: merge.

**Figure 7:**
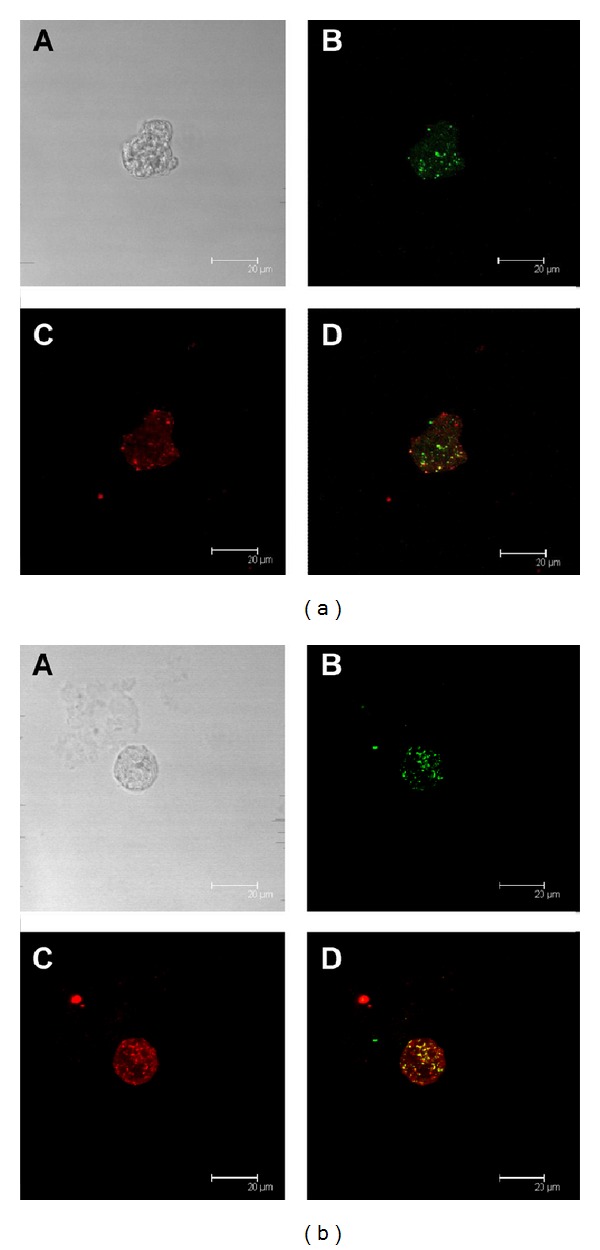
Colocalization of* Eh*CRT or* Ed*CRT and human C1q after interaction with NHS: human C1q and* Eh*CRT or* Ed*CRT colocalization estimated by confocal microscopy of (a)* E. histolytica*; (b)* E. dispar* trophozoites. Trophozoites were grown under axenic conditions and were allowed to adhere to sterile glass cover slips. Trophozoites were incubated with normal human serum (NHS) (source of C1q) and then fixed with 3.5% paraformaldehyde/PBS; thereafter, trophozoites were incubated with specific primary antibodies, rabbit anti-*Eh*CRT IgG and mouse anti-human C1q IgG, respectively. The reaction was revealed with Alexa Fluor 555 goat anti-rabbit IgG and Alexa Fluor 488 goat anti-mouse IgG. The micrographs show the maximal projection of the *z*-series. Scale bar represents 20 *μ*m. A: phase contrast microscopy; B:* Eh*CRT (red); C: C1q (green); D: merge.

To evaluate the interaction of* Eh*CRT/*Ed*CRT in trophozoites with human C1q, a colocalization assay was performed directly on trophozoites of both* E. histolytica* and* E. dispar* species by confocal microscopy. Both proteins clearly colocalized in previously permeabilized trophozoites; the fluorescent signal was detected in the cytoplasmic vesicles but was more concentrated near the cytoplasmic membrane (Figure 5); apparently, there are no differences in distribution of fluorescence in trophozoites between the two species. However, in nonpermeabilized trophozoites activated with peripheral mononuclear blood cells, the immunolocalization of CRT/C1q was detected on the surface membrane of trophozoites (Figure 6). Furthermore, when the trophozoites were incubated with NHS, the colocalization of both proteins was detected mainly in the cytoplasmic vesicles (Figure 7); this could be due to the destruction of trophozoites membranes induced by NHS.

### 3.5. Immunochemical Detection of* Eh*CRT and C1q in Amoebic Liver Abscess Lesions

**Figure 8:**
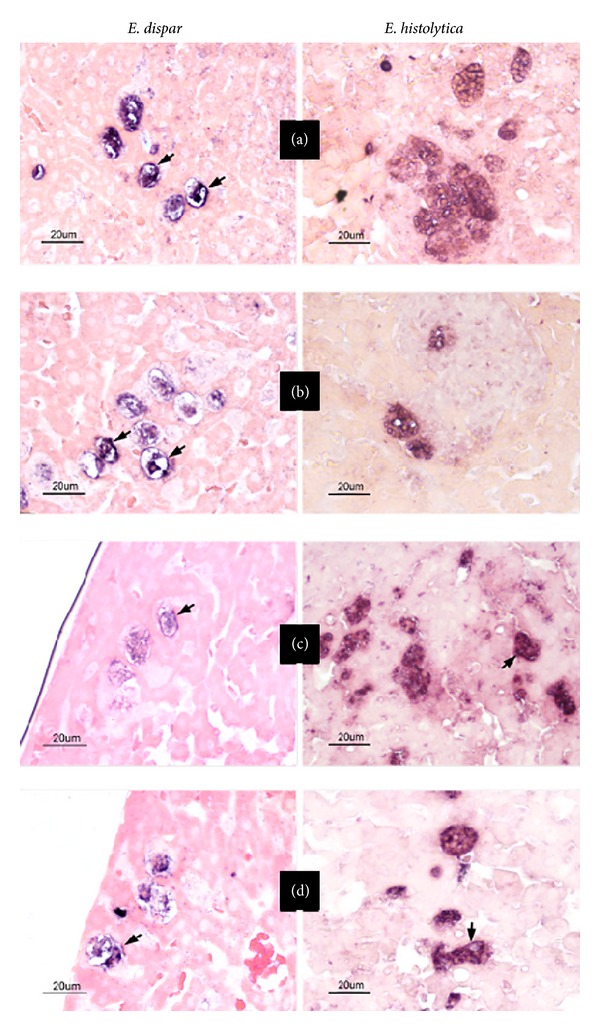
Immunohistochemical staining of* Eh*CRT or* Ed*CRT and C1q* in situ*. Representative images of immunohistochemical detection of CRT and C1q in amoebic liver abscess sections of livers from hamsters inoculated with* E. histolytica* HM1:IMSS virulent trophozoites and* E. dispar* SAW760 trophozoites and sacrificed at different times after inoculation for 30 min (a, b) and 3 h (c, d) (representative times). (a and c) Tissue section stained with mouse IgG against* Eh*CRT and (b and d) section stained with mouse IgG against C1q. Scale bar represents 20 *μ*m. The control assays of negative and secondary antibody were unstained; they are included as Supplementary Material.

Representative sections of hepatic tissue obtained at 30 min and 3 h after the intraportal inoculation of* E. histolytica* virulent trophozoites or* E. dispar* are shown in Figures 8(a) and 8(b), respectively. The immunodetection of CRT and C1q in the trophozoites established in the hepatic tissue is evident and displays a similar distribution on trophozoites as observed in the confocal microscopy assays (Figure 5). The immunohistochemical signals, both anti-*Eh*CRT and anti-C1q, were displayed in different size cytoplasmic vesicles. In some trophozoites, signals are apparently located on the cell surface membrane in both* Entamoebas*. It is clear that trophozoites apparently do not secrete or export the CRT protein into the hepatic tissue. The negative control and secondary antibody did not show background reactivity. These assays can be found in the Supplementary Material available online at http://dx.doi.org/10.1155/2014/127453.

### 3.6. Relative mRNA Quantification of* Ed*CRT and* Eh*CRT by qRT-PCR

**Figure 9:**
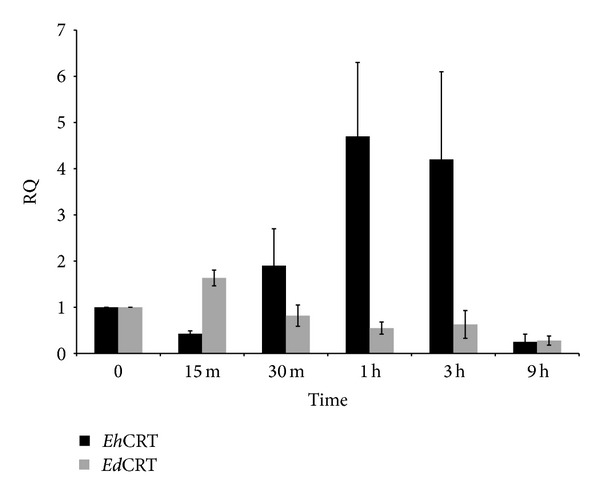
Relative quantification (RQ) of expression of mRNA for* Eh*CRT and* Ed*CRT. Reverse transcription real-time PCR was used to independently measure mRNA expression of* Ed*CRT and* Eh*CRT in trophozoites present in tissue sections of liver in hamsters after different times of postinoculation. The values represent the mean of three independent experiments. Differences between* E. histolytica* and* E. dispar* were compared through Student's *t*-test detecting statistical significance *(*P* = 0.005).

The relative quantification (RQ) of* Eh*CRT or* Ed*CRT mRNA expression is shown in Figure 9. The values correspond to the relative expression of cDNA into ALA specimens assayed by qPCR. The* Ed*CRT was expressed for a short period of time after inoculation (15 min RQ = 1.5) but it started to decline quickly for the remaining time resulting in lower levels than baseline. In contrast,* Eh*CRT increases at 30 min (RQ = 2) reaching a peak after 60 min (RQ = 5) between 3 h (RQ = 4.5); however, it decreased to values close to baseline thereafter. The level of expression of* Eh*CRT in comparison with* Ed*CRT was statistically significant (*P* = 0.04).

## 4. Discussion

*E. histolytica* and* E. dispar* are parasites whose natural host is the human being; their target organ is the large bowel. Therefore, they are in intimate contact with the local immune system. In human populations, the infection can be persistent or recurrent, self-limited, and usually asymptomatic (90% of cases), suggesting a balanced host-parasite interaction and some kind of immune response evasion mechanism*. E. histolytica* and* E. dispar* activate both classical and alternative pathways of serum complement [28, 29]. This activity is part of the immunological mechanisms induced by these protozoa during the early phases of host-parasite relationship. These mechanisms are among the main resistance mechanisms of hosts against parasite infection, but parasites have evolved alternative methods to evade immune attack and survive in the tissues of their host. One of these alternatives is the expression of an interesting and complex protein, calreticulin. This ubiquitous endoplasmic reticulum-associated protein has a large spectrum of functions, including the capacity to bind C1q, which is the first component of the classical serum complement pathway. CRT-C1q binding inhibits the activation of the complement cascade in different hosts; this mechanism has been considered an evasion mechanism of the immune response developed by a number of parasites [9, 10].

This evasion mechanism has been described in schistosomiasis, oncocercosis, trypanosomiasis [3, 5, 14], and now in amoebiasis. The interaction of CRT from* T. cruzi* (*Tc*CRT) with human C1q is one of the most studied systems. In this protozoan, CRT not only allows the evasion of the immune response but also modulates it. Furthermore, the presence of CRT on the surface of the parasite increases its infectivity by binding to C1q promoting the early phagocytosis of the parasite [30, 31].

In contrast to* T. cruzi*, in our* in vitro* assays,* E. histolytica* and* E. dispar* trophozoites do not express CRT on the external surface and do not export CRT into tissues in the* in vivo* model of amoebic liver abscess in hamsters [5]. Recently, the presence of CRT was reported in cytoplasmic membranes of trophozoites previously activated with Jurkat cells [17] or concanavalin A-activated trophozoites [32]. In the present study, we demonstrate that trophozoites activated by human PBMC also show the presence of CRT in the surface membrane (Figure 6). In the* in vivo* experiment of amoebic liver abscess in hamsters, the localization of CRT was also observed on the surface of trophozoites (Figure 8).

*Eh*CRT is highly immunogenic in humans, mice, and rabbits, suggesting that, in natural or experimental infection with* E. histolytica*,* Eh*CRT is in some way exposed to the host immunocompetent cells. The mechanism of this interaction may be time dependent and may occur via surface expression and/or by exposure to apoptotic or dead trophozoites as was previously reported [16].

In human trypanosomiasis, the role of* Tc*CRT as an immune evasion mechanism is easily understood because CRT is located on the surface of the trypomastigote in blood during the acute phase of infection and accessible to C1q. The role of* Entamoeba* CRT in pathogenicity is less clear. In intestinal amoebiasis, trophozoites are not totally exposed to complement system; apparently, the complement system only crosses to the mucosa membrane in conditions of disease as cancer, inflammatory bowel diseases, or autoimmune inflammatory intestinal diseases [33]. In amoebiasis infection, only in the case of invasive intestinal amoebiasis trophozoites are exposed to serum complement system. In the case of ALA, the trophozoites are indeed exposed to complement system. In this sense, trophozoites that express CRT on the surface membrane can bind C1q, induce the inhibition of the classical pathway of complement, and be protected from the lyses. This can be the case of our* in vivo* experimental model. During phagocytosis, C1q facilitates the binding to apoptotic epithelial cells by* E. histolytica* trophozoites [34]. Moreover,* Eh*CRT has been detected in the uropods induced in trophozoites by concanavalin A [32, 35], which may function as another mechanism by which* Eh*CRT/*Ed*CRT is exposed to the host immune system. However, capping formation in animal models and in human amoebic invasive lesions has not been observed. Draws attention the over expression of* Eh*crt gene in* E. histolytica* during first hours of host-parasite interaction in contrast with the* E dispar* specie, which do not over express this gene. This may suggest that over expression of crt gen could be a regulatory mechanisms that may allow the adaptation and survival of the parasite in the host tissues, as has been described in other parasites [31]. These are more suitable for the hostile environment in the liver. Besides, we cannot discard the possible selective pressure of serum complement over the infective trophozoites allowing the survival of complement resistant trophozoites in infected tissues. Finally, these trophozoites will be responsible for amoebic abscess development.

## Supplementary Material

In this section can you see, the negative control and secondary antibody of confocal microscopy for colocalization of *Eh*CRT and C1q in trophozoites of *E. histolytica* (figure 5-A). The negative control and secondary antibody of representative tissue slides of immunohistochemical detection of *Eh*CRT and C1q in amebic liver abscess sections of livers from hamster inoculated whit *E. histolytica* virulent (figure 8-A).
