# Supplementary material for: Entamoeba histolytica and E. dispar Calreticulin: Inhibition of Classical Complement Pathway and Differences in the Level of Expression in Amoebic Liver Abscess
Source: Biomed Res Int. 2014 Apr 22;2014:127453. doi: 10.1155/2014/127453 (PMC4016843; doi:10.1155/2014/127453)
Supplement: Supplementary file 1 — In this section can you see, the negative control and secondary antibody of confocal microscopy for colocalization of EhCRT and C1q in trophozoites of E. histolytica (figure 5-A). The negative control and secondary antibody of representative tissue slides of immunohistochemical detection of EhCRT and C1q in amebic liver abscess sections of livers from hamster inoculated whit E. histolytica virulent (figure 8-A). [file 127453.f1.pdf]

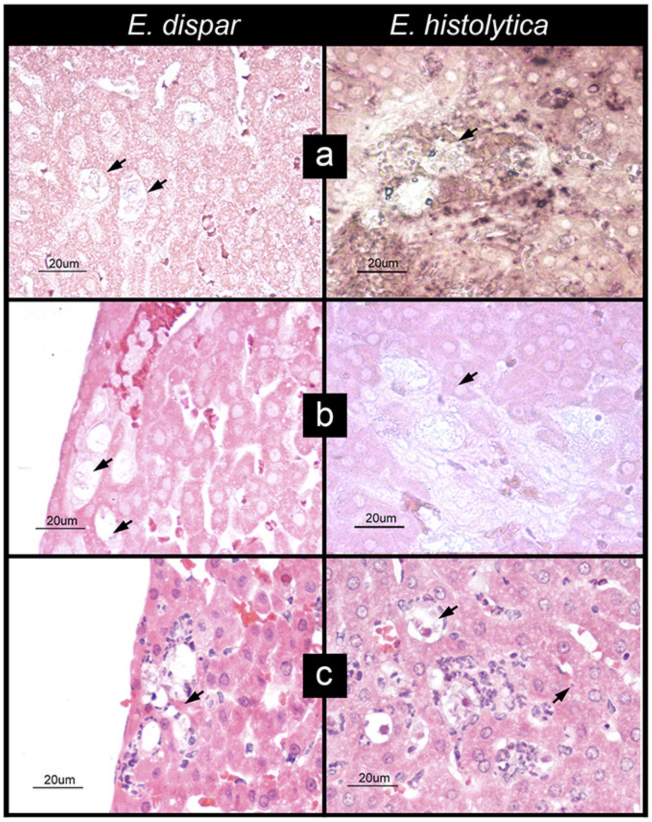

Figure 8A. . Immunohistochemical staining of *EhCRT* or *Ed*CRT and C1q in situ.

Representative images of immunohistochemical detection of CRT and C1q in amoebic liver abscess sections of livers from hamsters inoculated with*; E .histolytica* HM1:IMSS virulent trophozoites; *E. dispar* SAW760 trophozoites and sacrificed at different times after inoculation 30 min (a,) and 3 h (b); (a, b) Tissue sections stained with Monoclonal mouse IgG1 antibody against *Aspergillus niger* glucose oxidase was used as the negative control. c) hematoxylin/eosin stain. The arrows point out some demonstrative trophozoites. Scale bar represents 20 µm.

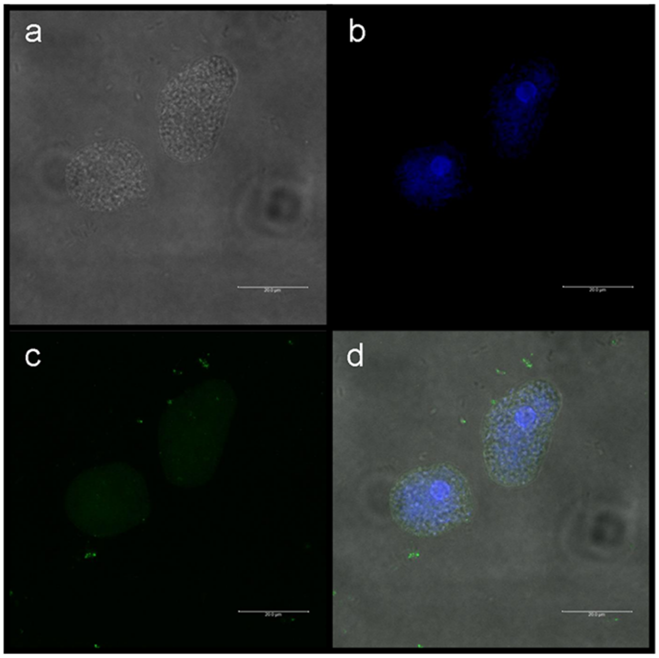

Figure 5A. Representative images of confocal microscopy assays: Co-location of *Eh*CRT or *Ed*CRT and human C1q. Here we shown the negative control when using *E. histolytica* trophozoites; a) Represent the differential interference contrast (DIC); b) preimmune mouse serum and trophozoite nucleus stained with DAPI; c) Trophozoites reacted with preimmune mouse serum and with anti-mouse Alexa-Fluor 488; d) Channel Mergin. The micrographs showed the maximal projection of the z-series. Scale bar represents 20 μm.
